# Identification of Genes Related to Paulownia Witches’ Broom by AFLP and MSAP

**DOI:** 10.3390/ijms150814669

**Published:** 2014-08-21

**Authors:** Xibing Cao, Guoqiang Fan, Minjie Deng, Zhenli Zhao, Yanpeng Dong

**Affiliations:** Institute of Paulownia, Henan Agricultural University, 95 Wenhua Road, Jinshui Area, Zhengzhou 450002, Henan, China; E-Mails: caoxibing2014@gmail.com (X.C.); dengmj1980@gmail.com (M.D.); zhaozl@gmail.com (Z.Z.); dongdyp@mail.nankai.edu.cn (Y.D.)

**Keywords:** *Paulownia tomentosa × Paulownia fortunei*, MMS, DNA sequence, DNA methylation, AFLP, MSAP, polymorphism, gene expression

## Abstract

DNA methylation is believed to play important roles in regulating gene expression in plant growth and development. Paulownia witches’ broom (PaWB) infection has been reported to be related to gene expression changes in paulownia plantlets. To determine whether DNA methylation is associated with gene expression changes in response to phytoplasma, we investigated variations in genomic DNA sequence and methylation in PaWB plantlets treated with methyl methane sulfonate (MMS) using amplified fragment length polymorphism (AFLP) and methylation-sensitive amplification polymorphism (MSAP) techniques, respectively. The results indicated that PaWB seedings recovered a normal morphology after treatment with more than 15 mg·L^−1^ MMS. PaWB infection did not cause changes of the paulownia DNA sequence at the AFLP level; However, DNA methylation levels and patterns were altered. Quantitative real-time PCR (qRT-PCR) showed that three of the methylated genes were up-regulated and three were down-regulated in the MMS-treated PaWB plantlets that had regained healthy morphology. These six genes might be involved in transcriptional regulation, plant defense, signal transduction and energy. The possible roles of these genes in PaWB are discussed. The results showed that changes of DNA methylation altered gene expression levels, and that MSAP might help identify genes related to PaWB.

## 1. Introduction

Epigenetic inheritance refers to phenotypic modifications in the absence of changes in the DNA sequence, and DNA methylation is one of the main epigenetic modifications. In plants, DNA methylation usually occurs at both CpG and CpNpG (where N = A, T or C) sites [1]. DNA methylation is associated with many biological processes, including transcriptional silencing of transgenes, regulation of gene expression, and genomic imprinting [2,3,4], and has become the focus of many studies. It was reported that DNA methylation regulated gene expression under biotic stress; for example, DNA methylation was found to induce post-transcriptional gene silencing in tomato infected with the Tomato yellow leaf curl Sardinia virus [5], and DNA methylation changed the transcript levels of genes in response to *Burkholderia phytofirmans* strain PsJN in infected potato plants [6].

Paulownia is one of the fastest growing shade trees in the world. The wood of this tree possesses several valuable characteristics, such as decay-resistance, strength, specific gravity, and fiber length, making it useful for furniture, pulp, and paper production. It has a prosperous root system, which makes it a good candidate for growing together with crops for efficient use of resources. Paulownia also can be used for biofuel and environment protection [7,8,9,10]. Paulownia witches’ broom (PaWB) caused by phytoplasma is the most destructive disease in Paulownia plantation regions. Paulownia infected by this disease developed a variety of symptoms including stunting, witches’ broom, yellowing of leaves and shorter internodes, and as a result the growth of the tree was seriously slowed down. It was estimated that PaWB affected 880,000 hectares of trees grown for timber production, corresponding to billions of dollars in economic losses [11,12]. Over the past 30 years, the relationship between PaWB morphological variations and physiological and biochemical changes became the focus of investigation. However, *PaWB* gene expression is still poorly understood, although whole transcriptome sequencing of *PaWB* and healthy paulownia plantlets has been carried out [13,14]. Recently, it was reported that PaWB plantlets recovered a healthy morphology after treatment with suitable concentrations of methyl methane sulfonate (MMS) [15,16]. It is well known that MMS can modify guanine and adenine to 7-methylguanine and 3-methyladenine, respectively, and that the phytoplasma can be removed in the infected plantlets treated with suitable concentration of MMS; however, the gene expression changes associated with the PaWB infection process and the molecular mechanism at DNA methylation level are not clearly understood. Therefore, the main aim of this study is to investigate the relation between DNA methylation and gene expression changes in PaWB plantlets, MMS-treated PaWB plantlets and healthy plantlets (HP) with methylation-sensitive amplification polymorphism (MSAP) technique. Moreover, the changes of gene expression levels were assessed through quantitative real-time PCR (qRT-PCR).

## 2. Results and Discussion

### 2.1. Morphological Changes of Paulownia Witches’ Broom (PaWB) Plantlets Treated with Methyl Methane Sulfonate (MMS)

**Figure 1:**
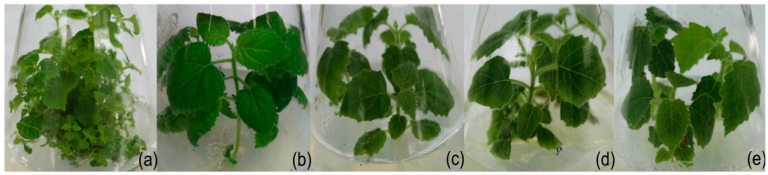
Changes of morphology in methyl methane sulfonate (MMS)-treated Paulownia witches’ broom (PaWB) plantlets. (**a**) PaWB plantlets; (**b**) 15 mg·L^−1^ MMS; (**c**) 30 mg·L^−1^ MMS; (**d**) 45 mg·L^−1^ MMS; (**e**) Healthy plantlets (HP).

**Figure 2:**
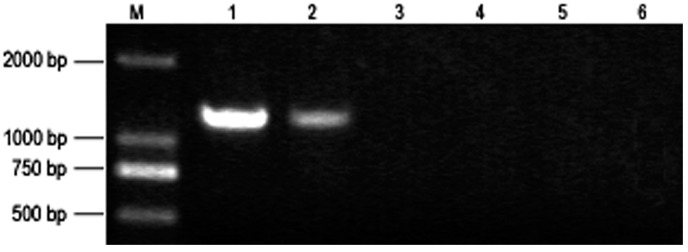
Detection of phytoplasma 16S rRNA in MMS-treated PaWB plantlets. **1**: PaWB plantlets; **2**: 15 mg·L^−1^ MMS; **3**: 30 mg·L^−1^ MMS; **4**: 45 mg·L^−1^ MMS; **5**: HP; **6**: double distilled water; **M**: DNA Marker.

The morphology of PaWB plantlets changed significantly after MMS treatment (Figure 1). The MMS-treated PaWB plantlets regained a healthy morphology (the small light yellow leaves without seta turned into green leaves with seta, and the short internodes changed into normal internodes). Both the PaWB plantlets treated with 30 and 45 mg·L^−1^ MMS changed into healthy morphology, while only very tiny axillary buds were found in the PaWB plantlets treated with 15 mg·L^−1^ MMS (Table S1). The rooting rates declined gradually with increasing MMS concentrations and the time of the first root was delayed after MMS treatment (*p* < 0.05). The phytoplasma (1.2-kb band) was not detected in plantlets treated with more than 15 mg·L^−1^ MMS (Figure 2). These results indicated that phytoplasma was removed in the PaWB plantlets which recovered a healthy morphology after treatment with MMS at appropriately adjusted concentrations.

### 2.2. DNA Sequence Polymorphism Revealed by Amplified Fragment Length Polymorphism (AFLP)

**Figure 3:**
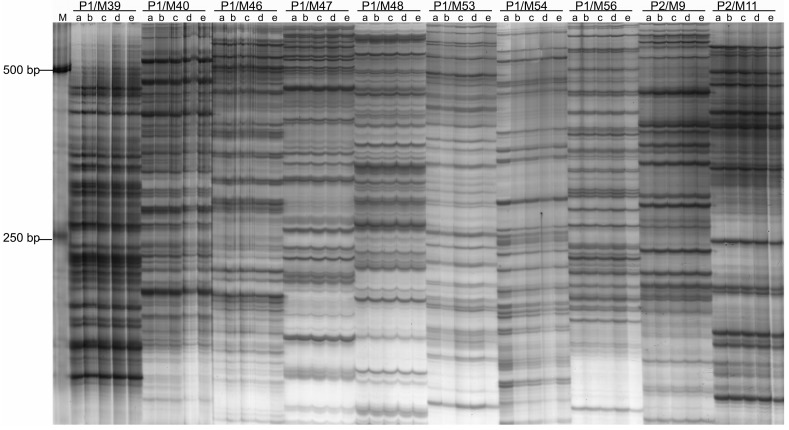
Amplified fragment length polymorphism (AFLP) amplification of MMS-treated PaWB plantlets. **a**: PaWB plantlets; **b**: 15 mg·L^−1^ MMS; **c**: 30 mg·L^−1^ MMS; **d**: 45 mg·L^−1^ MMS; **e**: HP; **M**: DNA Marker. P1/M39–P2/Mx: primer combinations.

An amplified fragment length polymorphism (AFLP) approach was used to analyze variations of the DNA sequences of MMS-treated PaWB plantlets and healthy ones. Ninety-six pairs of primer combinations failed to differentiate the DNA sequences of PaWB plantlets with phytoplasma from the MMS-treated PaWB plantlets and healthy ones, suggesting that the DNA sequences of those plantlets were not sensitive to MMS (Figure 3). No polymorphism band was observed in the DNA sequences of plantlets with phytoplasma and healthy ones using the same primer combinations. These results implied that the observed variations in the morphology of PaWB plantlets were not reflected in the DNA sequence at the AFLP level.

### 2.3. DNA Methylation Changes Revealed by Methylation-Sensitive Amplification Polymorphism (MSAP)

**Figure 4:**
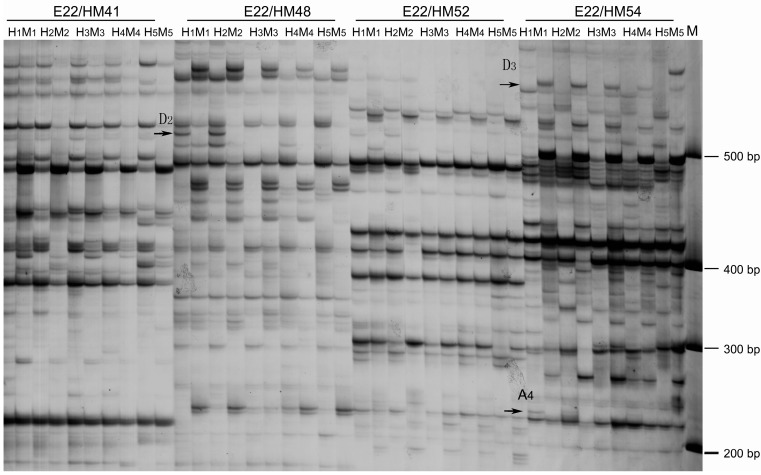
Changes of DNA methylation pattern in MMS-treated PaWB plantlets. **H1** and **M1**: band digested by *Eco*RI/*Hpa*II (H) and *Eco*RI/*Msp*I (M) in PaWB plantlets; **H2** and **M2**: band digested by H and M in plantlets with 15 mg·L^−1^ MMS treatment; **H3** and **M3**: band digested by H and M in plantlets with 30 mg·L^−1^ MMS treatment; **H4** and **M4**: band digested by H and M in plantlets with 45 mg·L^−1^ MMS treatment; **H5** and **M5**: band digested by H and M in HP. E22/HM41–E22/HMx: primer combination; **M**: marker. The arrows indicated 4 lanes of part of the methylation patterns variations only between PaWB plantlets and the ones with 15 mg·L^−1^ MMS treatment (H1, M1, H2, M2). D3, A4 and D2 indicated part of the methylation patterns variations. The band of D3, A4 and D2 were (0,1,0,1), (1,0,0,0) and (1,0,1,0), respectively.

The 96 pairs of primer combinations for MSAP were used to detect DNA methylation variations of the MMS-treated PaWB plantlets. The results showed that DNA methylation levels and patterns were different in those plantlets (Figure 4). The DNA methylation level of PaWB plantlets increased with increasing MMS concentrations (Table 1). The total methylated bands were 586, 692, 798 and 780 in plantlets treated with 0, 15, 30 and 45 mg·L^−1^ MMS, and the methylation levels were 28.15%, 31.85%, 33.86% and 35.21%, respectively (*p* < 0.05). However, the DNA methylation level of the PaWB plantlets treated with 45 mg·L^−1^ MMS was lower than that of the healthy ones (37.06%). For DNA methylation patterns, they were also different in the MMS-treated plantlets or healthy ones compared with the PaWB plantlets (Table 2), and these changes caused different DNA methylation status (Table 3). In the PaWB plantlets treated with 15, 30 and 45 mg·L^−1^ MMS, the DNA methylation polymorphism fragments were 342, 364 and 400, and the ratios of DNA methylation polymorphism were 15.92%, 18.33% and 19.46%, respectively. The DNA demethylation polymorphism fragments were 278, 290 and 312, and their ratios were 12.94%, 14.60% and 15.18%, respectively. These results indicated that DNA methylation was predominant in the MMS-treated PaWB plantlets. At the same time, in the HP, the ratio of the DNA methylation polymorphism was higher than that of the DNA demethylation polymorphism. These results suggested that the changes of DNA methylation levels and patterns might contribute to the observed variations in the morphology of PaWB and MMS-treated plantlets.

**Table 1:** Changes of DNA methylation level in MMS-treated PaWB plantlets. Total amplified bands = Bands of type I +Bands of type II +Bands of type III. Total methylated bands = Bands of type II + Bands of type III. Methylation level (%) = Total methylated bands/Total amplified bands × 100. The different letters within a column indicate significant difference, while the same letters within a column indicate no significant differences (*p* < 0.05).

MMS Concentration/(mg·L^–1^)	Total Amplified Bands	Bands of Type I	Bands of Type II	Bands of Type III	Total Methylated Bands	Methylation Level/%
0	2081	1495	199	387	586	28.15 ^a^
15	2173	1481	221	471	692	31.85 ^b^
30	2357	1559	267	531	798	33.86 ^c^
45	2215	1435	253	507	780	35.21 ^d^
HP	2193	1381	269	543	812	37.06 ^e^

**Table 2:** Changes of DNA methylation pattern in MMS-treated PaWB plantlets. H1 and M1: band digested by H and M in PaWB plantlets. Hx and Mx: band digested by H and M in treatments or HP. The first column presents the status of the DNA methylation bands of PaWB and MMS-treated plantlets or healthy ones. The second column presents the status of the DNA methylation base of PaWB and MMS-treated plantlets or healthy ones in the genome sequence. 1: presence of a band. 0: absence of a band. C and CC: cytosine methylation. **0**–**15**: number of DNA methylation patterns between 15 mg·L^−1^ MMS and PaWB plantlets; **0**–**30**: number of DNA methylation patterns between 30 mg·L^−1^ MMS and PaWB plantlets; **0**–**45**: number of DNA methylation patterns between 45 mg·L^−1^ MMS and PaWB plantlets; **0**–**HP**: number of DNA methylation patterns between healthy and PaWB plantlets.

Digestion				Changes of DNA Methylation Pattern		Number of Differences Bands				Pattern
H1	M1	Hx	Mx	PaWB	Treatments/HP	0–15	0–30	0–45	0–HP	
1	1	0	1	CCGG GGCC	CCGG GGCC	73	75	53	39	A_1_
1	1	1	0	CCGG GGCC	CCGG CCGG GGCC GGCC	65	83	69	67	A_2_
0	1	0	0	CCGG GGCC	CCGG GGCC	99	87	109	171	A_3_
1	0	0	0	CCGG CCGG GGCC GGCC	CCGG GGCC	105	119	169	159	A_4_
0	1	1	1	CCGG GGCC	CCGG GGCC	81	63	67	75	B_1_
1	0	1	1	CCGG GGCC	CCGG CCGG GGCC GGCC	39	55	61	53	B_2_
0	0	0	1	CCGG GGCC	CCGG GGCC	127	115	139	187	B_3_
0	0	1	1	CCGG GGCC	CCGG GGCC	31	57	45	109	B_4_
0	1	1	0	CCGG GGCC	CCGG CCGG GGCC GGCC	23	9	17	27	C
1	1	1	1	CCGG GGCC	CCGG GGCC	1145	1007	975	885	D_1_
1	0	1	0	CCGG CCGG GGCC GGCC	CCGG CCGG GGCC GGCC	81	71	97	81	D_2_
0	1	0	1	CCGG GGCC	CCGG GGCC	279	245	255	189	D_3_

### 2.4. Sequencing the MSAP Fragments

A set of 72 MSAP fragments that showed different DNA methylation or demethylation patterns, were isolated from the polyacrylamide gels and amplified by PCR. Single PCR products corresponding to 70 fragments (97.22%) were sequenced; two of the total isolated fragments (2.86%) did not give any PCR bands. A Translated Basic Local Alignment Search Tool (Blastx) search indicated that 20 fragments (27.78%) showed significant homology to known proteins with functions associated with plant defense, energy, transcription, protein biosynthesis, signal transduction and transport. However, the other 50 MSAP fragments (69.44%) showed no homology to sequences in the current GenBank databases (Table S2).

**Table 3:** Changes of DNA methylation status in MMS-treated PaWB plantlets. **Type A**: DNA methylation; **Type B**: DNA demethylation; **Type C**: uncertain DNA methylation; and **Type D**: DNA monomorphism. Total methylation bands = Bands of type A + Bands of type B + Bands of type C + Bands of type D. Type A (%) = Bands of type A/Total methylation bands × 100. Type B (%) = Bands of type B/Total methylation bands × 100. Type C (%) = Bands of type C/Total methylation bands × 100. Type D (%) = Bands of type D/Total methylation bands × 100.

Combination	Total Methylated Bands	Type A		Type B		Type C		Type D	
		Bands	Ratio/%	Bands	Ratio/%	Bands	Ratio/%	Bands	Ratio/%
0–15	2148	342	15.92	278	12.94	23	1.07	1505	70.07
0–30	1986	364	18.33	290	14.60	9	0.45	1323	66.62
0–45	2056	400	19.46	312	15.18	17	0.83	1327	64.54
0–HP	2042	436	21.35	424	20.76	27	1.32	1155	56.56

### 2.5. Quantitative Real-Time PCR (qRT-PCR) Analysis

**Figure 5:**
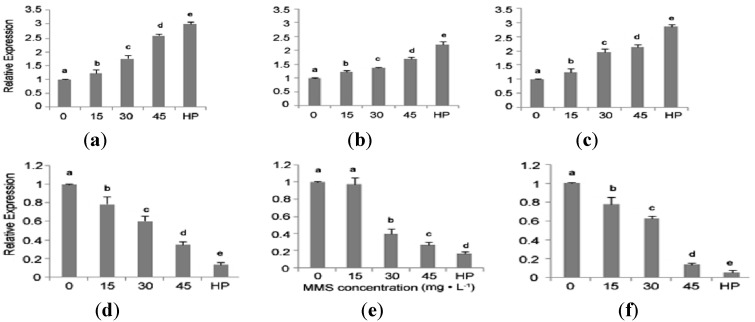
Expression levels of 6 different genes. (**a**) Relative expression of glutamyl-tRNA(Gln) amidotransferase subunit A; (**b**) Relative expression of photosystem II 47 kDa protein; (**c**) Relative expression of NADH: ubiquinone oxydoreductase subunit 7; (**d**) Relative expression of 26S protease regulatory subunit 6b; (**e**) Relative expression of xanthine dehydrogenase/oxidase; (**f**) Relative expression of zinc finger protein ZAT5. 0–45: MMS concentrations (mg·L^−1^). HP: Healthy plantlets. The different letters within a gene repression level indicate significant difference, while the same letters within a gene repression level indicate no significant differences (*p* < 0.05).

To investigate the effect of DNA methylation on gene expression, the expression level of six genes was confirmed by qRT-PCR. The results showed that three genes that encoded glutamyl-tRNA (Gln) amidotransferase subunit A, photosystem II 47 kDa protein and nicotinamide adenine dinucleotide (NADH): ubiquinone oxidoreductase subunit 7 were up-regulated in the PaWB plantlets treated with the higher MMS concentrations (Figure 5a–c); three genes that encoded 26S protease regulatory subunit 6b, xanthine dehydrogenase/oxidase and zinc finger protein ZAT5 were down-regulated (Figure 5d–f). These results were consistent with the changes of DNA methylation patterns found using MSAP, suggesting that MSAP is a reliable method to assess changes in gene expression.

### 2.6. Discussion

Phytoplasmas are difficult to culture outside their hosts in artificial culture media; therefore, to understand the mechanism of PaWB at the gene level, a comprehensive evaluation of the gene expression profiles is required. In this study, the relation between DNA methylation and gene expression was analyzed to gain insights into the mechanism of PaWB disease.

#### 2.6.1. Relation between PaWB and DNA Methylation

DNA methylation can result in stable epigenetic changes in gene activity without alteration of the DNA sequence [17]. In this study, the AFLP and MSAP approaches were used to identify the changes of DNA sequence and methylation in PaWB plantlets treated with different concentrations of MMS, respectively. In the AFLP analysis, 96 pairs of primer combinations failed to differentiate the DNA sequences of PaWB plantlets with phytoplasma from MMS-treated PaWB plantlets and healthy ones; the same bands were generated in these plantlets with the same pair of primers. This finding is in agreement with previous studies that analyzed at the simple sequence repeat (SSR) level [15,16]. It is important to note that the AFLP approach used in the present study has more discriminative power and is technically more demanding than SSR; thus, the AFLP results confirmed that variations in the phenotype of PaWB plantlets did not change the DNA sequence. In the MSAP analysis, the DNA methylation level of the morphologically healthy plantlets was higher than that of the PaWB plantlets.

This finding is in accordance with the results of an HPLC analysis [18]. These results indicated that the DNA methylation levels decreased in the plantlets infected by phytoplasma. Variations of the DNA methylation patterns were also detected in the MMS-treated PaWB plantlets and the healthy ones. Overall, the results with AFLP and MSAP demonstrated that the DNA sequences did not change among the PaWB plantlets and the ones treated with different MMS concentrations, but the DNA methylation levels and patterns did change. Further, the expression levels of six methylated genes related to PaWB varied significantly with changes of the morphology of the PaWB plantlets. Therefore, MSAP is an effective method for identifying genes involved in *Paulownia*–phytoplasma interactions, although only a fraction of the known DNA methylation polymorphisms were detected [5,19].

#### 2.6.2. DNA Methylation and Gene Expression Changes in PaWB

DNA methylation is a possible mechanism for the regulation of gene expression in plant development [20,21]. Substantial evidence supports a correlation between gene expression changes for DNA methylation and phytoplasma infection. In stolbur (isolate PO) phytoplasma-infected tomato [22], for example, the expression of some methylase and demethylase genes were found to be globally down-regulated. To our knowledge, the present paper is the first study to report alterations of DNA methylation result in changes of gene expression in PaWB. We identified six PaWB-related genes that were predicted to encode proteins involved in transcriptional regulation, plant defense, signal transduction and energy. The different expression level of these genes in diseased plantlets and healthy ones may be related to the occurrence of PaWB.

The gene encoding xanthine dehydrogenase/oxidase (XDH/XO) which was up-regulated in the PaWB plantlets, was reported to participate in abscisic acid (ABA) biosynthesis [13,23], this hormone influenced plant shoot growth, stem and internode elongation [13,24,25,26]. Moreover, ABA affects not only the stomatal aperture and decreases the photosynthetic CO_2_ assimilation rate, but also inhibits plant photosynthesis [10]. However, these effects might also result from reactive oxygen species (ROS) generated by XDH/XO. It was reported that ROS could damage cellular components, lipids, proteins and nucleic acid, and result in degradation of photosynthetic proteins [27]. The expression levels of two other PaWB-related genes predicted to encode NADH: Ubiquinone oxidoreductase subunit 7 and photosystem II 47 kDa protein were significantly down-regulated in the PaWB plantlets, which is in agreement with an earlier report in phytoplasma-infected mulberry, where the accumulation of ROS degraded the photosynthesis-associated proteins [28]. In addition, ROS and ABA together have been reported to play an important role in mediating signal transduction [29]. Thus, it can be speculated that XDH/XO is the key enzyme responsible for the reduction of photosynthesis-associated proteins in PaWB-infected plantlets. Another down-regulated gene encoding zinc finger protein ZAT5 can recognize cell signals (sugar, hormone, and pathogen) and activate plant defense pathways [30,31]. It was reported that ZAT5 could induce the host soybean calmodulin isoform-4 (*GmCaM-4*) gene in response to pathogen infection [32]. Therefore, this protein might take part in a complex signal network in the paulownia plantlets’ response to PaWB.

Phytoplasma are heavily restricted to the phloem tissues of plants; therefore, successful colonization of phytoplasma requires nutrients such as carbohydrates, nitrogen, phosphate, proteins, amino acids and ATP from their host phloem parenchyma cells [28,33,34]. In this study, two genes encoding glutamyl-tRNAGln amidotransferase and 26S protease regulatory subunit 6b are probably involved in the phytoplasma metabolic pathways. The qRT-PCR analysis showed that the expression level of the gene encoding glutamyl-tRNAGln amidotransferase was very low in the PaWB plantlets. Glutamyl-tRNAGln amidotransferase was reported to be confined to biosynthesis of asparagine, which was a good source of nitrogen for microorganism growth [35,36]. Its low expression level may lead to nitrogen starvation and nitrogen metabolism disorder in paulownia tissues, and inhibit the synthesis of amino acids, proteins, hormones, chlorophyll II and other essential metabolic constituents, resulting in the morphology of PaWB plantlets with stunting and yellowing leaves [28,37,38]. The 26S protease is reported to be the major non-lysosomal protease, and is an integral component of the plant protein ubiquitin-proteasome system (UPS) degradation pathway where it plays an important role in plant defense by altering the proteome [39,40]. It was reported that pathogens could hijack the UPS pathway to counteract the plant’s defense system or to harness the host UPS to target host proteins for degradation to improve their virulence in the host [41,42]. Moreover, UPS as a regulating system may involve sucrose and hormone signal transduction [43,44,45]. These results suggested that DNA methylation decreases gene expression levels in PaWB plantlets.

## 3. Experimental Section

### 3.1. Plantlets, MMS Treatments, and Morphological Changes

The healthy and PaWB plantlets of *Paulownia tomentosa × Paulownia fortunei* were obtained from the Institute of Paulownia, Henan Agricultural University, Zhengzhou, China. The obtained plantlets were cultivated on 1/2 Murashige-Skoog (MS) medium [46] for 30 days, then, the uniformly terminal buds at 1.5-cm long were cut from the PaWB plantlets and transferred into 1/2 MS medium (40 mL) containing 0, 15, 30, or 45 mg·L^−1^ MMS in 100 mL flasks supplemented with 25 mg·L^−1^ sucrose and 8 mg·L^−1^ agar (Sangon, Shanghai, China). The terminal buds from the HP were collected in the same way and transferred into the supplemented 1/2 MS medium (40 mL) without MMS. All plantlets were cultured in the dark at 20 °C for 5 days, then cultured at 25 ± 2 °C and 130 μmol·m^−2^·s^−1^ light intensity on a 14 h light/10 h dark photoperiod for 25 days. For each MMS treatment, every three terminal buds were planted in one flask. Each treatment was carried out in triplicate as described above. Morphological changes of the plantlets were observed on the 10th, 20th, and 30th day during their cultivation in MMS medium. The method used was according to Fan, *et al.* [47]. After 30 days, the terminal buds that were 1.5-cm long and were in good condition were harvested from all the plantlets, and immediately frozen in liquid nitrogen and stored in at −80 °C.

### 3.2. PaWB Phytoplasma Detection

Total genomic DNA from the terminal buds was isolated using the cetyl trimethyl ammonium bromide (CTAB) (Beijing Chemical Co., Beijing, China) method according to Zhang, *et al.* [48]. The RNase (Invitrogen, Carlsbad, CA, USA) was used to ensure the DNA was free of genomic RNA contamination. The PaWB phytoplasma DNA was amplified by nested-PCR using the R_16_mF_1_/R_16_mR_1_ and R_16_mF_2_/R_16_mR_2_ primers [49]. The method of PCR amplification and agarose gel electrophoresis were according to Fan, *et al*. [47].

### 3.3. AFLP and MSAP Analyses

AFLP analysis was performed as described by Cao, *et al.* [50]. *Pst*I and *Mse*I (Li-COR, Co., Lincoln, NE, USA) restriction enzymes were used in the digestion. The PCR products were separated on 6% sequencing gels and then visualized by silver staining. The adapters and primers used for AFLP are shown in Table S3. The MSAP analysis was performed as described by Cao, *et al.* [51]. Two restriction enzyme combinations *Eco*RI/*Msp*I (TakaRa, Dalian, China) and *Eco*RI/*Hpa*II (TakaRa Dalian, China) were used in the digestion, respectively. The PCR products were separated on 4% sequencing gels, and the AFLP and MSAP methods of polyacrylamide gel electrophoresis were carried out as described by Cao, *et al*. [50]. The adapters and primers used for MSAP are shown in Table S4.

### 3.4. Band Scoring and Data Analysis

After silver staining, a statistical analysis was constructed based on the bands obtained from AFLP and MSAP. Only clear and reproducible bands were scored, where the presence of a band was scored as “1” and the absence of a band was scored as “0”. For the MSAP analysis, the results were scored based on the presence or absence of a band in the products of the *Eco*RI/*Hpa*II (H) and *Eco*RI/*Msp*I (M) digestions. Three types of DNA methylation were possible on the electrophoresis gels: no methylation (Type I), where the band was present in both H and M; hemi-methylation (Type II), where the band was present in H but absent in M; full-methylation (Type III), where the band was absent in H but present in M. Two kinds of DNA methylation patterns were considered: Methylation polymorphism, and monomorphism. Three types of DNA methylation polymorphisms were: DNA methylation (Type A), DNA demethylation (Type B), and uncertain DNA methylation (Type C). Among the Type A polymorphisms, (A_1_ and A_2_) was DNA de novo methylation, where the band was present in both H and M in the untreated PaWB plantlets, but only in H or M in the MMS-treated plantlets or healthy ones; and (A_3_ and A_4_) was DNA hypermethylation, where the band was present only in H or M in the untreated PaWB plantlets, but was absent in both H and M in the MMS-treated plantlets or healthy ones. On the contrary, Type B (B_l_, B_2_, B_3_ and B_4_) was DNA demethylation, where the bands of (B_l_ and B_2_) were opposite to the bands of (A_1_ and A_2_), the bands of (B_3_ and B_4_) were opposite to the bands of (A_3_ and A_4_). Type C was uncertain DNA methylation, where the types of DNA methylation bands could not be determined between the untreated PaWB plantlets and the MMS-treated plantlets. The monomorphisms were Type D (D_1_, D_2_ and D_3_), where the bands were present/absence in M and H in the untreated PaWB plantlets were same to the bands in MMS-treated PaWB plantlets or healthy ones. The following equations were used to calculate the various characteristics as follows: Total DNA methylation level (%) = [(Type II + Type III)/(Type I + Type II + Type III)] × 100; Total DNA polymorphism bands = A + B + C + D; DNA methylation polymorphisms (%) = [(A + B + C)/(A + B + C + D)] × 100; DNA methylation monomorphisms (%) = [D/(A + B + C + D)] × 100.

### 3.5. Sequencing of the MSAP Fragment

The clean and reproducible methylated fragments were excised from the gel with a clean razor blade. The bands on the gel slices were recovered using a polyacrylamide gel kit (Sangon). The DNA fragments were reamplified with the same primers under the same conditions used for selective amplification (Table S4). The PCR products were cloned in a pMD18-T vector (TakaRa, Dalian, China) and sequenced. The positive clones were sequenced with M13 primers by automatic sequencing. The advanced Standard Nucleotide: Blastn and Blastx programs on the NCBI website [52] were used for homology searches of the cloned DNA sequences that gave quality-reads against the GenBank nucleotide and protein sequence databases.

### 3.6. Quantitative Real-Time PCR Analysis

Total RNAs were isolated using an Aidlab Total RNA Extraction Kit (Aidlab, Beijing, China). Then RNase-free DNase I (Invitrogen) was used to ensure the genomic DNA was free of RNA contamination. cDNA synthesis was performed using an iScript cDNA Synthesis Kit (Bio-Rad) following the manufacturer’s instructions. QRT-PCR was conducted with gene-specific primers, which were designed using Primer premier 6.0 (PREMIER Biosoft International, Palo Alto, CA, USA), and synthesized commercially (Invitrogen), each reaction tube contained 10 μL SsoFast supermix (Bio-Rad), 0.4 μM forward primer (Invitrogen), 0.4 μM reverse primer (Invitrogen), and 1 μL cDNA in a final volume of 20 μL. The reaction was carried out for 40 cycles using the following conditions: 95 °C for 1 min, 95 °C for 10 s, and 55 °C for 15 s. The reactions were run on a CFX96TM Real-Time PCR Detection System (Bio-Rad). Relative quantification of gene expression was calculated by the 2^–ΔΔ*C*t^ method and normalized to 18S rRNA. Each sample was performed in triplicate. The SPASS 19.0 software (SPASS, Inc., Chicago, IL, USA) was used to analysis the statistical significance. The primers for the qRT-PCR are listed in Table S5.

## 4. Conclusions

This study showed that PaWB plantlets treated with a minimum of 15 mg·L^−1^ MMS recovered healthy plantlets, in which 1.2 kb bands specific to phytoplasma could not be detected with nested-PCR. However, the PaWB plantlets treated with less than 15 mg·L^−1^ MMS did not change morphologically. The DNA sequences of the plantlets remained the same at the AFLP level, with or without phytoplasmas, but the DNA methylation levels and patterns were altered when determined by MSAP. Moreover, six gene expression levels were tightly related to DNA methylation changes of the PaWB plantlets, indicating that the relationship between DNA methylation and gene expression might clarify the mechanism of PaWB. This information may also provide a basis for studies into the role of DNA methylation in other plant–pathogen interactions.

## Supplementary Files

Supplementary File 1
