# Supplementary material for: Identification of Genes Related to Paulownia Witches’ Broom by AFLP and MSAP
Source: Int J Mol Sci. 2014 Aug 21;15(8):14669–83. doi: 10.3390/ijms150814669 (PMC4159874; doi:10.3390/ijms150814669)
Supplement: Supplementary File 1 [file ijms-15-14669-s001.pdf]

Supplementary Information

Table S1. Changes of the development of MMS-treated PaWB plantlets. The different letters within a column indicate significant difference, while the same letters within a column indicate no significant differences ($p < 0.05$).

MMS Concentrations /(mg·L^{-1})	Rooting Ratio/%			Rooting Time/Days	Axillary Crowns	Leaves and Internodes	Terminal Crown Growth
	10 Days	20 Days	30 Days				
0	90.0 ^{a}	100 ^{a}	100 ^{a}	6	Yes	Small light yellow leaf without seta and short internodes	Expand
15	76.7 ^{b}	90.0 ^{a}	100 ^{a}	7	Yes	Green leaf with seta and normal internodes	Normal
30	67.2 ^{c}	90.0 ^{a}	100 ^{a}	8	None	Green leaf with seta and normal internodes	Normal
45	36.1 ^{d}	85.0 ^{b}	88.3 ^{b}	9	None	Green leaf with seta and normal internodes	Normal
HP	100 ^{a}	100 ^{a}	100 ^{a}	5	None	Green leaf with seta and normal internodes	Normal

Table S2. MSAP fragments homologous to those in the databases.

Function	Gene Name [a]	Sequence Homology [b]	Accession No.	Positives/(%)	Expect
Protein biosynesis and destination	Yb1	Glutamyl-tRNA(Gln) amidotransferase subunit A	XP_003599919.1	85.19	3.00×10^{-5}
	Yb2	30S ribosomal protein S5	NP_180936.1	87.18	8.00×10^{-12}
	Yb3	30S ribosomal protein S5	NP_180936.1	87.18	9.00×10^{-12}
	Yb4	30S ribosomal protein S5	NP_180936.1	87.18	8.00×10^{-12}
	Yb5	Dihydrodipicolinate synthetase	YP_004039809.1	97.44	1.00×10^{-19}
	Yb6	Dihydrodipicolinate synthetase	YP_004039809.1	100	5.00×10^{-19}
Transport	Yb7	Ribosome export/assembly protein 1	Q12019.1	91.67	4.00×10^{-5}
	Yb8	Cation proton exchanger	XP_004245552.1	70.73	4.00×10^{-4}
	Yb9	Cation proton exchanger	XP_002297994.1	70.73	1.00×10^{-5}
	Yb10	Cation proton exchanger	XP_002297994.1	70	4.00×10^{-4}
Energy	Yb11	Photosystem II 47 kDa protein	BAL44657.1	100	2.00×10^{-6}
	Yb12	NADH:ubiquinone oxydoreductase subunit 7	X86706.1	97	2.00×10^{-35}
	Yb13	NADH:ubiquinone oxydoreductase subunit 7	X86706.1	97	2.00×10^{-35}
Signal transduction	Yb14	Chase2 sensor protein	YP_004040123.1	100	8.00×10^{-9}
Transcription	Yb15	Transcription factor HB29	Q9SEZ1.1	88.89	4.00×10^{-8}
Defense	Yb16	26S protease regulatory subunit 6b	XP_002523664.1	96.15	3.00×10^{-14}
	Yb17	Xanthine dehydrogenase/oxidase	XP_003597436.1	93.1	2.00×10^{-11}
	Yb18	Xanthine dehydrogenase 2	NP_195216.2	100	5.00×10^{-11}
	Yb19	Zinc finger protein ZAT5	XP_004246607.1	92.86	3.00×10^{-8}
	Yb20	ZF-HD homeobox protein At4g24660	XP_002273802.1	93.75	6.00×10^{-11}

[a], The methylated fragments were obtained from MMS-treated PaWB and healthy plantlets; [b], The sequence information was obtained from the GenBank database.

Table S3. AFLP adapters and primers used in this study. P1/M1–P64/M64 are numbers of selective-amplification primer combinations.

**Name**	**Sequence (5'-3')**				
Adaptors	*Pst* I-F CTCGTAGACTGCGTACATGCA *Pst* I-R TGTACGCAGTCTAC *Mse* I-F GACGATGAGTCCTGAG *Mse* I-R TACTCAGGACTCAT				
Pre-amplification primer	*Pst* I (P) GACTGCGTACATGCAG *Mse* I (M) GATGAGTCCTGAGTAA				
	P+AAA/M+AAA(P1/M1)	P+AAC/M+AAG(P1/M2)	P+AAA/M+AAC(P1/M3)	P+AAA/M+AAT(P1/M4)	P+AAA/M+ACC(P1/M11)
	P+AAA/M+ACT(P1/M12)	P+AAC/M+ATA(P1/M13)	P+AAA/M+ATG(P1/M14)	P+AAA/M+ATC(P1/M15)	P+AAA/M+ATT(P1/M16)
	P+AAA/M+GAG(P1/M18)	P+AAT/M+GAC(P1/M19)	P+AAA/M+GGA(P1/M21)	P+AAA/M+GGG(P1/M22)	P+AAA/M+GGC(P1/M23)
	P+AAA/M+GCA(P1/M25)	P+AGA/M+GTG(P1/M30)	P+AAA/M+GTC(P1/M31)	P+AAA/M+CAA(P1/M33)	P+AAA/M+CAC(P1/M35)
	P+AAA/M+CGC(P1/M39)	P+AGG/M+CGT(P1/M40)	P+AAA/M+CTG(P1/M46)	P+AAA/M+CTC(P1/M47)	P+AAA/M+CTT(P1/M48)
	P+AAA/M+TGA(P1/M53)	P+AGG/M+AGG(P1/M54)	P+AAA/M+TGT(P1/M56)	P+AAG/M+ACA(P2/M9)	P+AAG/M+ACC(P2/M11)
	P+AAG/M+ACT(P2/M12)	P+AAG/M+ATC(P2/M15)	P+AAA/M+GAA(P2/M17)	P+AAG/M+GGA(P2/M21)	P+AAG/M+GGG(P2/M22)
	P+AAG/M+GGC(P2/M23)	P+AAG/M+GCA(P2/M25)	P+AAG/M+GCT(P2/M28)	P+AAG/M+GTT(P2/M32)	P+AAG/M+CAC(P2/M35)
	P+AAG/M+CAT(P2/M36)	P+AAG/M+CTG(P2/M46)	P+AAG/M+CTC(P2/M47)	P+AAG/M+TAA(P2/M49)	P+AAG/M+TTT(P2/M64)
Selective-amplific-ation	P+AAC/M+AGA(P3/M5)	P+AAC/M+AGC(P3/M7)	P+AAC/M+ACT(P3/M12)	P+AAC/M+ATA(P3/M13)	P+AAC/M+ATG(P3/M14)
primer com-binations	P+AAC/M+GAA(P3/M17)	P+AAC/M+GAT(P3/M20)	P+AAC/M+GCG(P3/M26)	P+AAC/M+TAG(P3/M50)	P+AAC/M+TGG(P3/M54)
	P+AAC/M+TCA(P3/M57)	P+AAC/M+TCC(P3/M59)	P+AAC/M+TCT(P3/M60)	P+AAC/M+TTC(P3/M63)	P+AAT/M+GCA(P4/M25)
	P+AAT/M+GTG(P4/M30)	P+AAT/M+CTC(P4/M47)	P+AAT/M+CTT(P4/M48)	P+AAT/M+TAC(P4/M51)	P+AAA/M+AGA(P5/M5)
	P+AGA/M+ACA(P5/M9)	P+AGA/M+ATA(P5/M13)	P+AGA/M+ATC(P5/M15)	P+AGA/M+ATT(P5/M16)	P+AGA/M+GTG(P5/M30)
	P+AGA/M+CAG(P5/M34)	P+AGA/M+CAT(P5/M36)	P+AGA/M+CCA(P5/M41)	P+AGA/M+CTA(P5/M45)	P+AGA/M+CTG(P5/M46)
	P+AGA/M+CTT(P5/M48)	P+AGA/M+TAA(P5/M49)	P+AGA/M+TAT(P5/M52)	P+AGA/M+TGC(P5/M55)	P+AGA/M+TCC(P5/M59)
	P+AGA/M+TCT(P5/M60)	P+AGA/M+TTA(P5/M61)	P+AGG/M+AAA(P6/M1)	P+AGG/M+ACA(P6/M9)	P+AGG/M+ATG(P6/M14)
	P+AGG/M+ATT(P6/M16)	P+AGG/M+GTT(P6/M32)	P+AGG/M+TAC(P6/M51)	P+AGG/M+TCA(P6/M57)	P+AGG/M+TCT(P6/M60)
	P+AGG/M+GGA(P8/M21)	P+AGG/M+CAA(P8/M33)	P+AGG/M+TAT(P8/M52)	P+AGG/M+TCA(P8/M57)	P+TTC/M+TTC(P63/M63)
	P+TTT/M+TTT(P64/M64)				

Table S4. MSAP adapters and primers used in this study. Selective-amplification primer combinations are each of the *Eco*RI primer combined with each of the *Hpa*II/*Msp*I primer.

**Name**	**Sequence (5'-3')**			
	***Eco*RI**		***Hpa*II/*Msp*I**	
Adaptor	Adaptor-F	CTCGTAGACTGCGTACC	Adaptor-F	GATCATGAGTCCTGCT
	Adaptor-R	AATTGGTACGCAGTCTAC	Adaptor-R	CGAGCAGGACTCATGA
Pre-amplification primer	*Eco*RI (E)	GACTGCGTACCAATTCA	*Hpa*II/*Msp*I (HM)	ATCATGAGTCCTGCTCGGT
Selective-amplification primer	E+AAA (E1)		HM+AAC (HM4)	
	E+AGG (E11)		HM+ATT (HM6)	
	E+TAC (E20)		HM+ACA (HM13)	
	E+TTT (E22)		HM+TGG (HM27)	
	E+TGA (E25)		HM+GTC (HM40)	
	E+TGT (E26)		HM+GGA (HM41)	
	E+GAC (E36)		HM+GCC (HM48)	
	E+CGC (E60)		HM+CAC (HM52	
			HM+CTT (HM54)	
			HM+CTC (HM56)	
			HM+CGG (HM59)	
			HM+CCT (HM62)	

Table S5. Primers used for quantitive real-time PCR analysis.

Gene Name	Forward Sequence (5'-3')	Reverse Sequence (5'-3')
18S	ACATAGTAAGGATTGACAGA	TAACGGAATTAACCAGACA
Yb1	GCAGATATGGAAACCAAGTT	CATACCTCAGAACCAAACC
Yb11	CTCGGTCAGTTGCTTTCC	ATACAGAGCCATCGAGCC
Yb12	GGAGGCCAGGTCATTTGA	CACGCACTTCCCTTTACTC
Yb16	CGACTATGCCATTATTCTG	GTGAAGCGGATACAATCT
Yb17	CCACTTGTGAGCAGAATC	AGATCGAAGGGGCATTTG
Yb19	AATTTGTGGGTCGGAGTT	AGTCCTGGTACTAGCACTA
